# Study of anaemia in pregnancy and low birth weight in fluoride endemic area of Western Rajasthan: a cohort study

**DOI:** 10.12688/f1000research.134720.2

**Published:** 2024-03-19

**Authors:** Pankaj Bhardwaj, Neha Mantri, Nitin Kumar Joshi, Praveen Suthar, Praveen Sharma, Purvi Purohit, Pratibha Singh, Shashank Shekhar, Shweta Khandelwal, Giribabu Dandabathula, Pankaja Ravi Raghav, Nikky Ramawat, Sanjeev Jain, Manoj Patil, Abhay Gaidhane, Zahiruddin Quazi Syed, Deepak Saxena

**Affiliations:** 1 School of Public Health, All India Institute of Medical Sciences,, Jodhpur, Rajasthan, India; 2 Department of Community Medicine and Family Medicine, All India Institute of Medical Sciences, Jodhpur, Rajasthan, India; 3 Department of Biochemistry, All India Institute of Medical Sciences, Jodhpur, Rajasthan, India; 4 Department of Obstetrics and Gynecology, All India Institute of Medical Sciences, Jodhpur, Rajasthan, India; 5 Regional Remote Sensing Centre – West, National Remote Sensing Centre,, Indian Space Research Organization, Jodhpur, Rajasthan, India; 6 State Health Department, Government of Rajasthan, Jaipur, Rajasthan, India; 7 Community Medicine, Jawaharlal Nehru Medical College, Datta Meghe Institute of Higher Education and Research, Wardha, Maharashtra, India; 8 Adjunct Faculty, Datta Meghe Institute of Higher Education and Research, Wardha, Maharashtra, India; 9 Epidemiology, Indian Institute of Public Health, Gandhinagar, Gujarat, India

**Keywords:** Newborns, Infant, Pregnancy, Fluoride, Low Birth Weight, Anemia, Geographic Information System

## Abstract

**Background:**

Fluoride is a noxious element known to destroy gastrointestinal mucosa, leading to erythrocytes’ destruction and causing anaemia. The birth weight of newborn babies is a significant indicator of a child’s vulnerability to the risk of childhood diseases and chances of existence.

**Methods:**

This prospective cohort study was planned to find linkages between fluorosis and the low-birth weight of newborn babies with anaemic mothers. Antenatal mothers until the 20th week of gestation were followed up till delivery in the Antenatal Clinic of a District Hospital in one of the known fluoride-endemic districts (Nagaur) and the other not-so-endemic district (Jodhpur) of Western Rajasthan.

**Results:**

Around 19% of the newborn in Jodhpur and around 22% in Nagaur had low birth weight. Mean fluoride values in water samples were measured to be 0.57 (range from 0.0 to 2.7 PPM) in Jodhpur and 0.7 (range from 0.0 to 3.4 PPM) in Nagaur.

**Conclusions:**

Thus, in fluoride endemic areas, other factors should be included besides iron and folic acid supplementation for improving anaemia in pregnant women. This calls for assessing the effectiveness of de-fluoridation activities along with the area’s most common indigenous food practices.

## Introduction

Maternal and neonatal mortality rates are high in developing countries ( Beck *et al.*, 2010). Low birth weight (LBW) is the most common cause of neonatal mortality ( Eshete *et al.*, 2019; Watkins *et al.*, 2016). The birth weight of newborn babies is a significant indicator of a child’s vulnerability to the risk of childhood diseases and chances of existence ( Alemayehu *et al.*, 2020). The World Health Organization (WHO) defines LBW as weight at birth less than 2.5 kg (“WHA Global Nutrition Targets 2025: Low Birth Weight Policy Brief,” n.d.). LBW may be an outcome of either preterm birth or retarded fetal growth. According to a report by UNICEF on “The State of the World’s Children 2008”, the highest percentage (43%) of LBW babies below five years of age are in India. The prevalence of LBW babies in South Asia is 42%, in developed countries 35%, in developing countries 26% and 25% in the rest of the world ( Sachdev, 2001).

India has been documenting the serious problem of anaemia in pregnancy, resulting in LBW ( Batista Filho *et al.*, 2008; Rahman *et al.*, 2016; World Health Organization, 2015). The Ministry of Health and Family Welfare, Government of India, has launched the National Anaemia Prophylaxis Programme to combat anaemia. The decision to supplement iron along with folic acid (iron (60 mg) and folic acid (500 μg) orally for 90 days) to pregnant women visiting antenatal clinics (ANCs) during the first and second trimesters was implemented across the country. The Indian Council of Medical Research (ICMR) conducted a study in 11 Indian states in 1985–86 and discovered that the intervention did not affect pregnant women’s haemoglobin (Hb) levels. This resulted in the power of iron and folic acid tablets increasing to 100 mg and 500 mg, respectively, which has been popular since 1992 ( Susheela *et al.*, 2010).

However, despite so many interventions to reduce anaemia during pregnancy, expected results have not so far been obtained ( Beard, 2000; Kapil *et al.*, 2019; Vijayaraghavan *et al.*, 1990). So, another factor needs to be investigated, and one such factor is fluoride intake. Fluorosis, an underestimated and sometimes debilitating condition, is being caused by high levels of fluoride in food and contaminated groundwater in India ( Bello, 2020). Fluoride causes serious destruction to the gastrointestinal mucosa by destroying microvilli resulting in non-absorption of nutrients from the regimen ( Das *et al.*, 1994; Dhar and Bhatnagar, 2009; Susheela *et al.*, 2018). Fluoride also destroys erythrocytes, thereby contributing to the loss of Hb and anaemia. Interestingly, fluoride exposure during pregnancy was not significantly associated with fertility or birth outcomes, especially among women living in areas with levels of fluoride consistent with water fluoridation ( Goodman *et al.*, 2022). A study conducted in the United States discovered that greater water fluoride concentrations were connected with a 3.3-month earlier age of menarche ( Malin *et al.*, 2022). Further, cognitive skills in older adults living in high fluoride drinking water locations may be compromised ( Ren *et al.*, 2021). A south Indian researcher discovered that nutritional status has an effect on the severity of fluorosis, with impoverished children being more affected by severe fluorosis ( Mahantesha *et al.*, 2016).

Fagin’s report on fluoride’s second thoughts in 2008 is a warning to all concerned, as he exposed the possibility of fluoride-producing diseases in the teeth, bone, brain, and thyroid gland ( Fagin, 2008). Fluoride is a toxic substance that promotes thyroid hormone production in children when it is consumed during the intrauterine growth stage ( Gedalia *et al.*, 1964). As a result, the thyroid hormone status of married women before conception may need to be examined ( McNeil and Stanford, n.d.).

Fluorides, like other elements, have both good and harmful properties that have a substantial impact on public health. Even though it is noted that an optimal dose of 1mg of fluoride per litre in drinking water is beneficial for the prevention of tooth decay, extended exposure to greater concentrations can lead to adverse effects on teeth and bones ( Azami-Aghdash *et al.*, 2013). A concentration of fluoride higher than 2 mg/l causes teeth corrosion to increase with fluoride intake, and this can occur in conjunction with other conditions or might increase certain risks ( Susheela, 2013). In pregnant women, placental carriage of fluorides happens as early as the 19 ^th^ week of gestation ( Gedalia *et al.*, 1964). Studies in a few countries have demonstrated linkages between dental fluorosis and LBW. Contrastingly, other literatures have found increased risk of adverse birth outcomes in areas where fluoride levels in drinking water are high (> 1.5 mg/L) ( Goyal *et al.*, 2020). However, there is a paucity of studies in the Indian context correlating exposure to fluoride and anaemia in mothers resulting in LBW infants ( Guth *et al.*, 2020). Therefore, this study was planned to find linkages between fluorosis, anaemia during pregnancy, and outcome regarding the birth weight of newborn babies.

## Methods

### Study design and study setting

For years, Rajasthan has been one of the worst states impacted by fluorosis, which has had a significant impact on the health of the native people. According to assessment from the State Institute of Health and Family Welfare in 2004, all 32 districts of Rajasthan are fluoride endemic areas. From June 2017 to September 2018, a prospective cohort study was done in the Antenatal Clinics of a District Hospital in one of the known fluoride-endemic areas (Nagaur) and one not so endemic district (Jodhpur) in Western Rajasthan. The reporting was done in accordance with the checklist for Strengthening the Reporting of Observational Studies (STROBE) ( Patil, 2023a).

### Study participants

All pregnant women until the 20 ^th^ week of gestational age attending the ANC clinics from the month of initiation of study were enrolled for the study. Participants were enrolled based on consecutive sampling as they appear in the Out Patient Department (OPD). All participants were given a brief about the objectives of the study and were given choices for participation. After obtaining written informed consent, pregnant women were followed-up till delivery for estimating the birth weight of the new-born. For new-borns, consent was obtained from the parent/guardian. Those who did not give consent were excluded from the cohort. All participants were recruited till January 2018 and a ticket with the unique line listing number was provided to them with the dates for their next visit. The follow-up for each participant was conducted in the 2 ^nd^ and 3 ^rd^ trimester during their routine ANC check-up at the hospital and repeat test (Hb test, test for water fluoride and urinary fluoride) was conducted. To minimize loss-to-follow up, telephonic reminder was made to the enrolled participants and visits were planned according to their convenience. After the delivery, the new-born was enrolled and birth weight was estimated.

### Ethical considerations

Ethical approval was obtained from the Institutional Ethics Committee of All India Institute of Medical Sciences, Jodhpur, Rajasthan, India on 20 ^th^ August 2016 (Ref. AIIMS/RES (04)/2017/169). All participants were asked to sign an informed consent before enrolment.

### Study tool

Data was captured using a semi-structured questionnaire designed to elicit information on socio-demographic details, medical history, obstetric history, vital parameters, 24-hour diet, source of drinking water, medication history, dental and skeletal fluorosis ( Patil, 2023b). The tool was pilot tested and refined based on experiences of the 20 participants who were not the part of the study. Appropriate measures were followed to minimize the potential bias at recruitment by providing a unique line listing number and concealing the identification details from the samples.

***Haemoglobin test*** – For the purpose of the study, we used the WHO Criteria for Anaemia as reference for pregnant women (pregnant women with haemoglobin levels less than 11.0 g/dl in the first and third trimesters and less than 10.5 g/dl in the second trimester were considered anaemic). A point-of-care instrument Haemocue 201+ analyser (Manufactured at HeamoCue AB, Sweden, Catalogue no. 1627013065)was used to measure haemoglobin level at three times – at enrolment, second visit and third visit. The trained research staff were appointed for conducting the procedure. All necessary PPE measures were used to avoid contamination during the procedure. After cleaning the puncture site with an alcohol swab (middle finger or ring finger of the either hand), a prick was made on the fingertip using a needle and 2–3 drops of blood was taken on a microcuvette and loaded in the analyser. The haemoglobin concentration was then displayed as a digital reading, in either g/dl or mmol/l in 15–45 seconds. Results were also mentioned in the patient summary sheet and necessary consultation with obstetrician was arranged if any intervention was required.

***Fluoride test*** (water and urine) – All participants were given a 500 ml water sample sterile high-density polyethylene bottle for collecting water from their residence at the time of enrolment in the study. All enrolled participants were given a sterile polyethylene urine container (50 ml) by adding 0.2% Etheylenediaminetetraacetic acid (EDTA) [Orion Manufacturer] to bottles before collection during the visit to collect urine and were stored in refrigerator at 4°C to avoid bacterial contamination. The patient identification was concealed and all samples were labelled with unique line listing number and transported to the central laboratory for a test within 48 hours. Water and urine analysis was carried out in the biochemistry research unit, according to analytical protocol ( NIOSH Manual of Analytical Methods, P&CAM, 2 ^ND^ edition) using a Thermo Scientific Fluoride Ion Meter Model (Ion 85 Analyser Radiometer Copenhagen). As per NIOSH manual, Fluoride Ion is used as an analyte in Ion selective electrode (ISE) technique. A Fluoride standard was prepared and an electrode (Orion, 9609BNWP) was setup using TISAB III (Orion, 940911). Then, the urine sample was diluted with equal volume of TISAB III. The electrode was dipped in the sample and the reading was displayed on the screen. The instrument was calibrated with the reference electrode each time before running samples in a batch of 60–70 samples. The results were noted in the log book simultaneously with the line listing number.

### Sample size calculation

We enrolled a total of 1440 pregnant women in the study. As per the reference article by Aditya *et al.* ( Arun *et al.*, 2022), Mean ± Std. Deviation of Water Fluoride (mg/L) in Low-birth-weight population = 0.54 ± 0.36.

Using the formula for minimal Sample size required-

$$\;N=\frac{{\left(Z_{1-\alpha /2}\right)}^{2}\;\sigma^{2}}{d^{2}}$$

Where $Z_{\alpha }$ = 1.96 at a 5% level of significance

$$\text{Estimated Error}=4\%\;\text{of Mean}={\left(0.54/100\right)}^{*}5=0.02.$$

$$\text{Minimum sample size required was}=N=\frac{{\left(1.96\right)}^{2}\;{\left(0.36\right)}^{2}}{{\left(0.0216\right)}^{2}}=1245$$

Assuming a non-response of 10% and rounding off, the sample size was finalized to 1440.

### Statistical analysis

Microsoft Excel v.2016 (Microsoft ^®^ Corp., Redmond, WA) was used to enter the data and was analysed using Statistical Packages for Social Sciences (SPSS) version 23 software (IBM Corp., Armonk, NY). The descriptive statistics (mean, percentages) were used to summarize quantitative data for the study outcomes. Chi-squared test was used to examine the differences between categorical variables and was considered significant when a p-value < 0.05. Further, to provide the graphical presentation, spatial mapping of the various variable like level of fluoride in urine and low birth weight in both the district was done using Arc GIS v 10.3( “ArcGIS 10.3,” n.d.).

## Results

Out of 1440 pregnant women enrolled, 50% (n = 720) were from the Jodhpur district, and 50% (n = 720) were from the Nagaur district of western Rajasthan ( Patil, 2023c, 2023d). The mean age of women from Jodhpur was 25.51 (±3.87) years, and from Nagaur were, 23.74 (±3.85) years. The mean Hb (gm%) level in pregnant women in the Jodhpur district was 10.4 gm% as compared to the Nagaur district (9.1 gm%).

### Fluoride content in water and urine samples of Nagaur and Jodhpur district

Mean fluoride values in water samples were measured to be 0.57 (range from 0.0 to 2.7 PPM) in Jodhpur and 0.7 (range from 0.0 to 3.4 PPM) in Nagaur.

**Figure 1.**
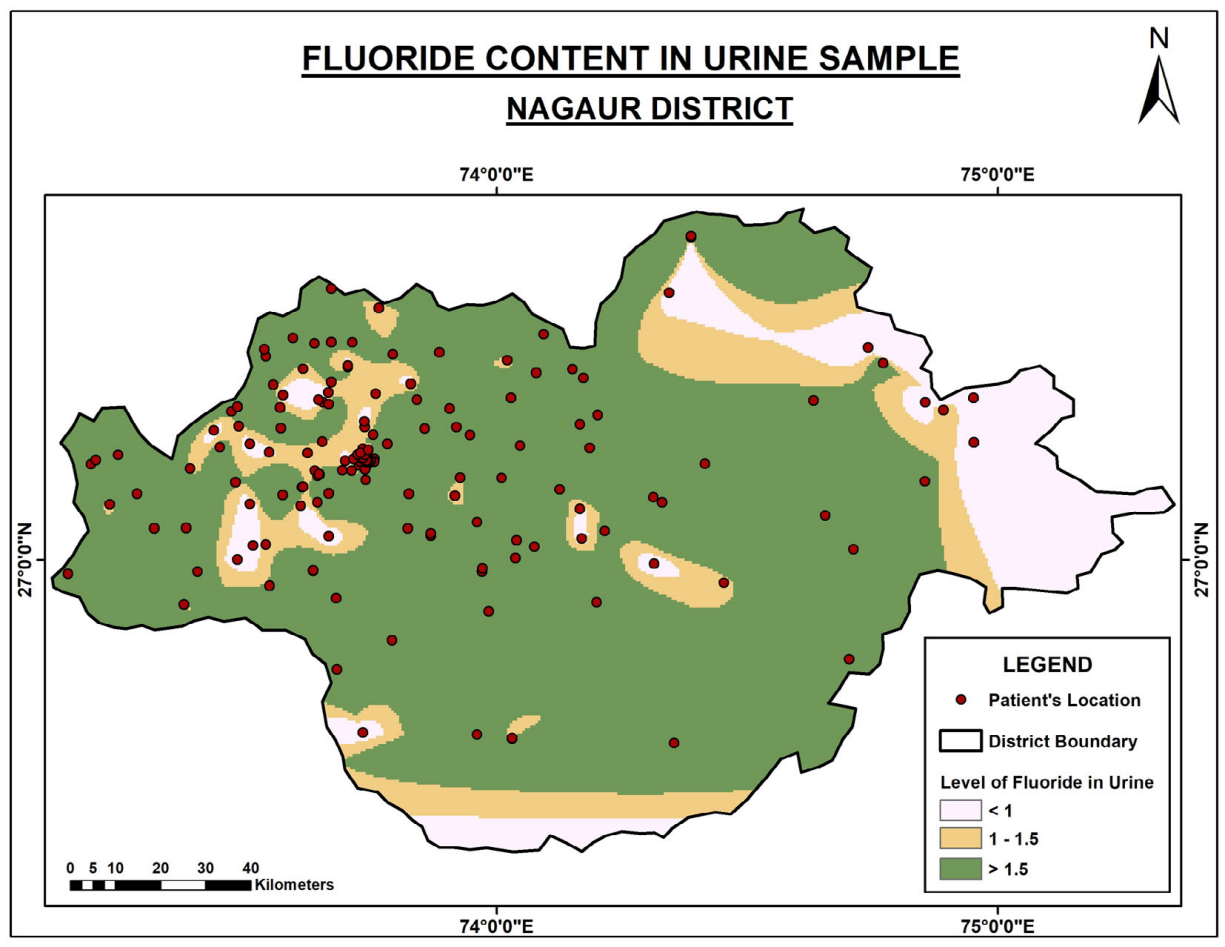
Fluoride content in urine samples of Nagaur District.

**Figure 2.**
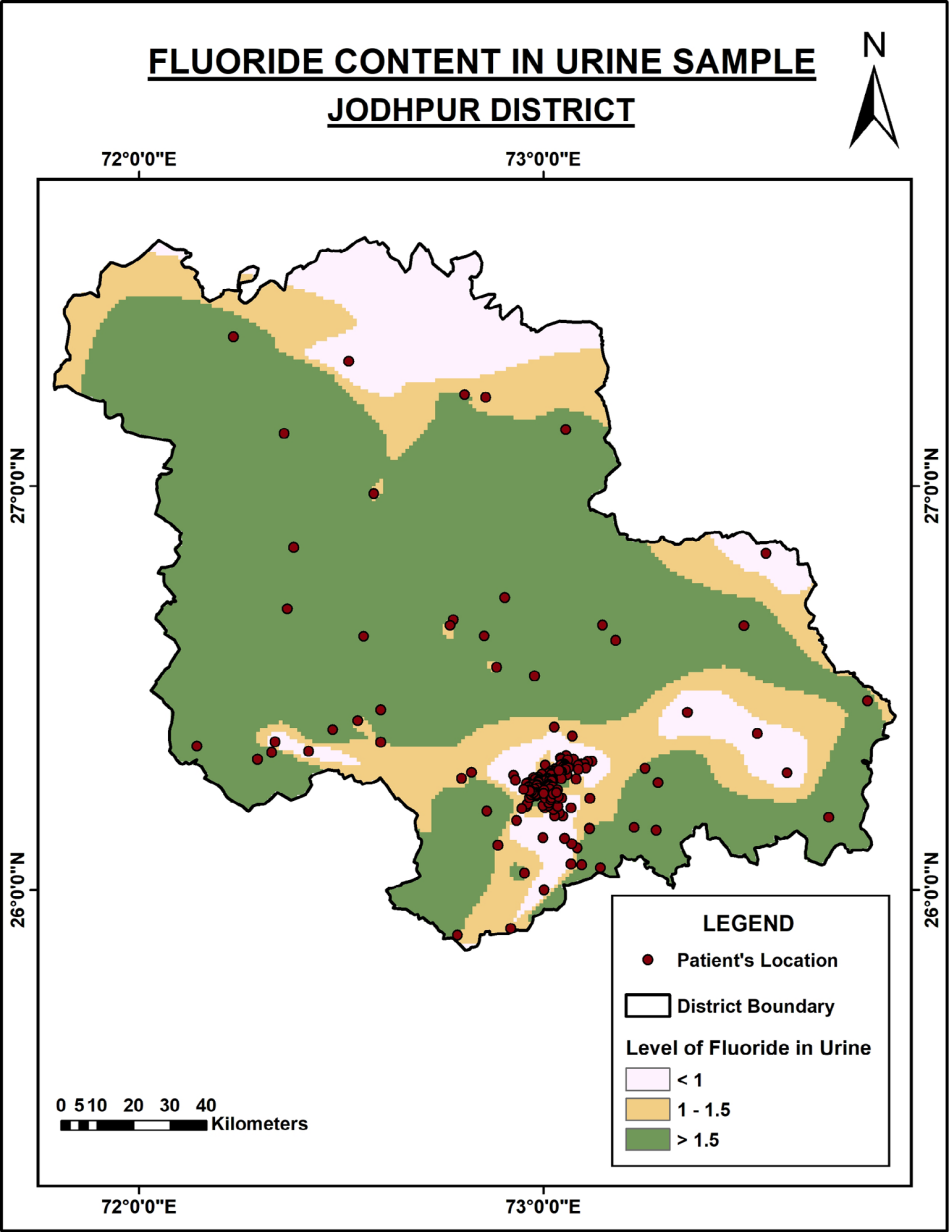
Fluoride content in urine samples of Jodhpur District.

The excretion of mean fluoride value in the urine of pregnant females was found to be 1.4 (range from 0.0 to 12.7 mg/L) in the Jodhpur district and 2.2 (range from 0.1 to 13.7 mg/L) in the Nagaur district. Figure 1 depicts the urinary fluoride content in the Nagaur district, which is very high on the northern side. Figure 2 describes the level of urinary fluoride in the Jodhpur district. The level of fluoride is low compared to the Nagaur district. However, in the district’s central and southern regions, fluoride content is high in the urine as it is excessive in the groundwater.

### Low birth weight babies in Nagaur and Jodhpur district

The mean weight of newborns from the Jodhpur district was 2.8 kg (±0.56), and from Nagaur was 2.8 kg (±0.44). Around 19% of the newborns in Jodhpur and around 22% in Nagaur had LBW. However, there was no significant difference in LBW babies in both districts (p=0.151).

**Figure 3.**
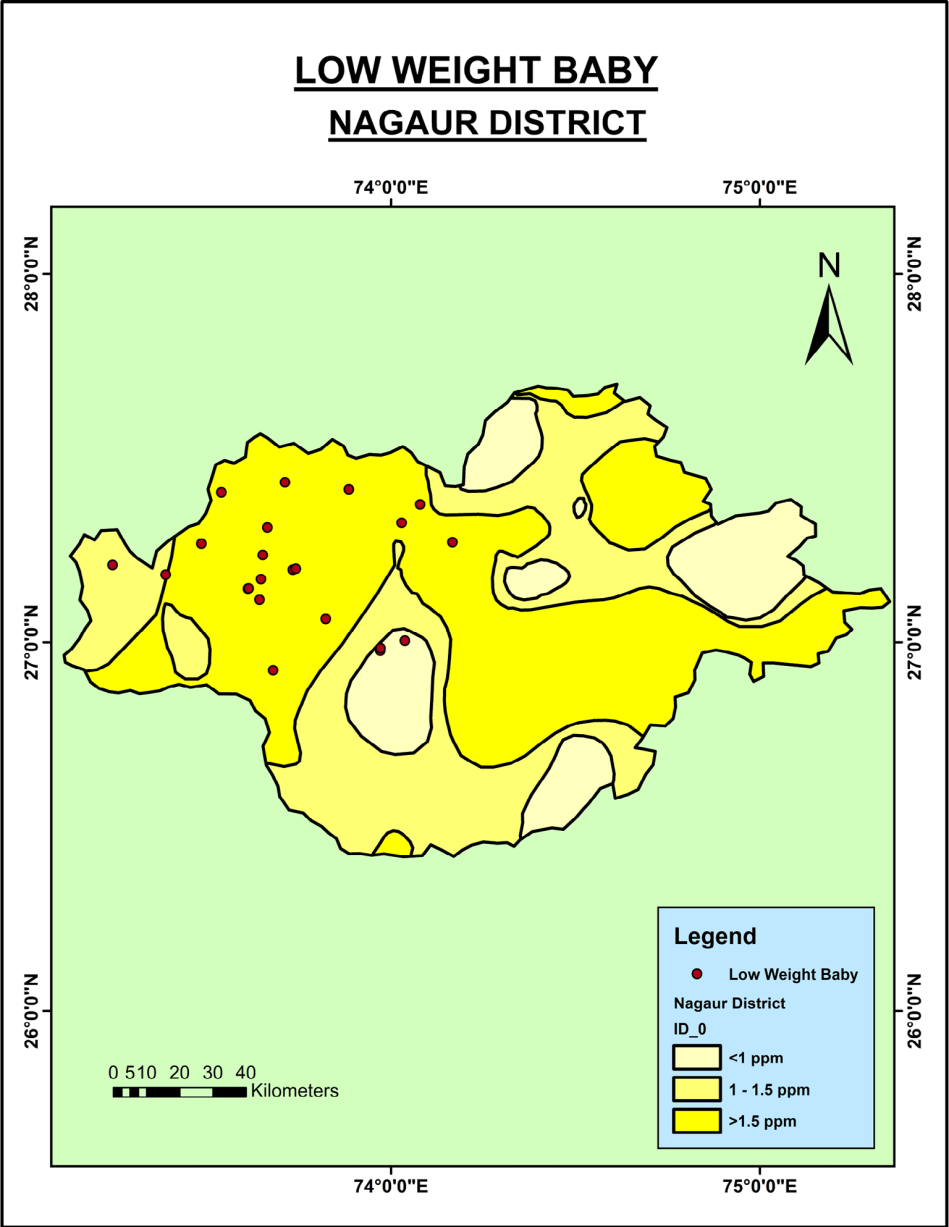
Low birth weight babies in Nagaur District.

**Figure 4.**
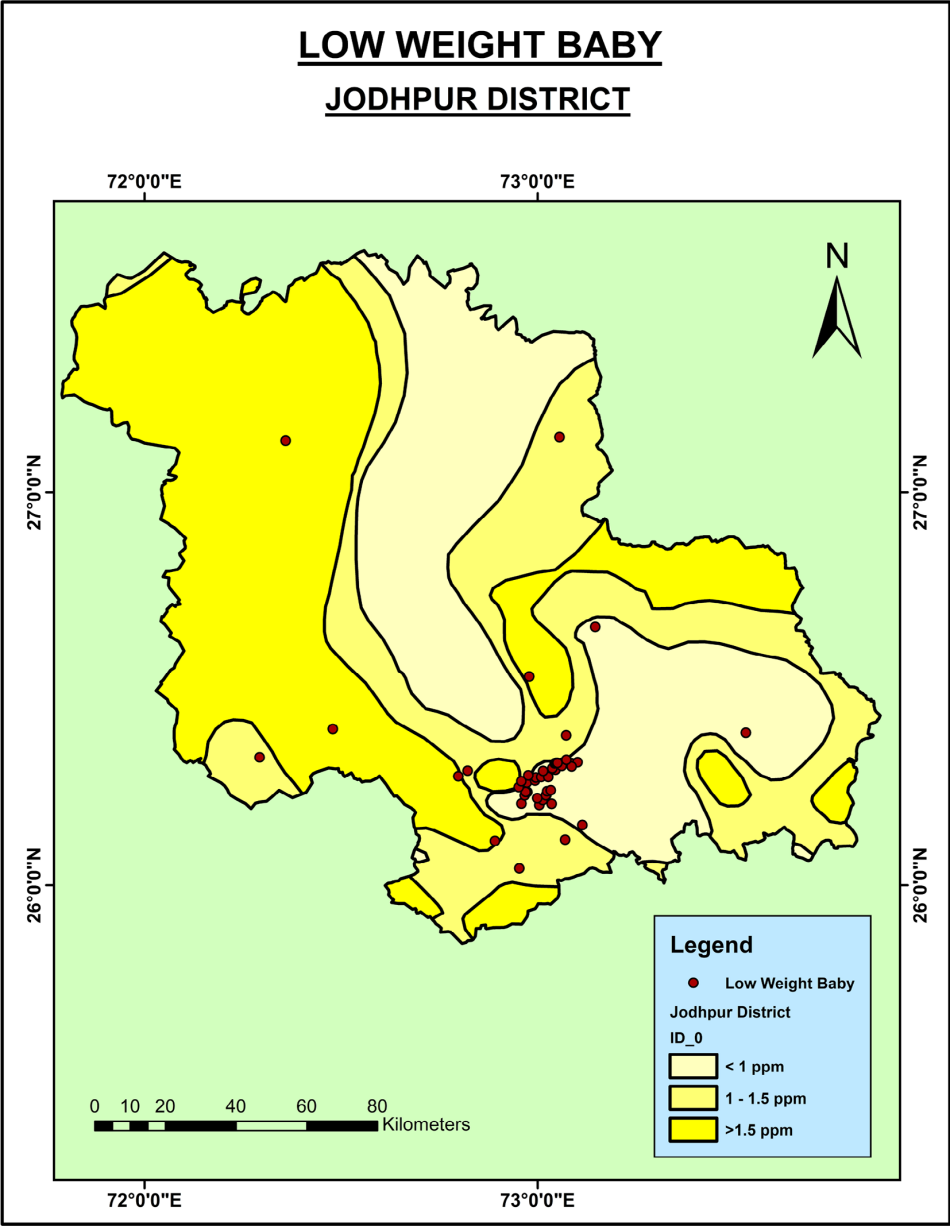
Low birth weight babies in Jodhpur District.

As shown in Figure 3, the LBW babies were seen in the regions of the Nagaur district where the groundwater fluoride level was very high. However, in the context of the Jodhpur district ( Figure 4), the low weight babies were seen in the southern part of the district where the groundwater fluoride level is low. This might be because of the consumption of the fluoride from the other sources.

Table 1 illustrates the socio-demographic characteristics of western Rajasthan’s Jodhpur and Nagaur fluoride endemic districts. A significant association was seen between fluoride level in the water (p = 0.00001), Body Mass Index (BMI) (p = 0.00001), anaemia (p = 0.00001), and dental fluorosis (p = 0.00001). Table 2 illustrate the association of fluoride in water with BMI, anaemia and dental fluorosis among pregnant women.

**Table 1.** Socio-demographic and selected characteristics of fluoride endemic districts.

Characteristics	Jodhpur District	Nagaur District
	(n = 720)	(n = 720)
Mean age of pregnant women (years)	25.51 (+3.87)	23.74 (+3.85)
Mean weight of pregnant women (kg)	62.7 (+11.53)	58.6 (+10)
Mean height of pregnant women (cm)	161.9 (+7.52)	163.1 (+3.62)
**Socio-economic Status (%)**		
Upper Class	17.92	0.69
Upper Middle Class	55.56	1.67
Middle Class	12.22	4.87
Lower Middle Class	11.94	87.64
Lower Class	2.36	5.13
**Literacy Status (%)**		
No Schooling	2.8	16.4
Primary Education	27.5	58.2
Secondary Education	21.1	19.4
Graduation	28.2	5.6
Post-Graduation	20.4	0.4
**Birth Weight (%)**		
Low Birth Weight	18.89	22.08
Normal Birth Weight	81.11	77.92
Water Fluoride (%)	1.67	7.08
Dental Fluorosis (%)	9.02	23.33
Chin Test (%)	5.13	12.63
Stretch Test (%)	4.16	7.5
Muscular Weakness (%)	20.14	38.33
Tingling Sensation (%)	12.5	37.22

**Table 2.** Association of fluoride level in water with BMI, anemia, and dental fluorosis.

Variables		Water Fluorosis			χ ^2^	p-value
		Optimal n = 939 (%)	Sub-Optimal n = 438 (%)	High n = 63 (%)		
Birth Weight	Low	207	80	8	5.0727	0.07915
	Normal	732	358	55		
Anaemia	No	191	152	9	37.0404	0.00001
	Yes	748	286	54		
Dental Fluorosis	No	417	247	17	28.1077	0.00001
	Yes	522	191	46		
Chin Test	No	860	401	51	8.3956	0.01502
	Yes	79	37	12		

A significant association was also seen in dental fluorosis with birth weight and anaemia (p < 0.005). Table 3 illustrates dental fluorosis’s association with baby birth weight (normal birth weight (NBW) and LBW) and anaemia in pregnant women (no anaemia and anaemia). The excretion of fluoride in pregnant women’s urine and haemoglobin (Hb) has a statistically significant linear relationship. The direction of the relationship is negative, i.e., urine fluorosis and haemoglobin of pregnant women are negatively correlated. This illustrated that greater fluoride excretion is associated with lower haemoglobin levels (r = -0.2).

**Table 3.** Association of dental fluorosis across baby birth weight and anemia status of pregnant women.

Variables		Dental Fluorosis		χ ^2^	p-value	Relative Risk (RR)
		No Fluorosis (%)	Fluorosis (%)			
Birth Weight	Normal Birth Weight	754 (46.31)	874 (53.69)	19.1402	0.000012	RR = 1.63 (CI 1.27-2.09)
	Low Birth Weight	46 (28.40)	116 (71.60)			
Anaemia	No anemia	160 (34.26)	307 (65.74)	74.1282	0.00001	RR = 2.2 (CI 1.84-2.63)
	Anaemia	206 (15.57)	1117 (84.43)			

Urine fluoride and newborn birth weight have a statistically significant linear relationship. The direction of the relationship is negative, i.e., urinary fluoride and newborn birth weight are negatively correlated. This illustrated that greater urinary fluoride is associated with birth weight (r = -0.01). The relative risk of LBW was 1.63 times more in high fluorosis cases. Also, the relative risk of anaemia among pregnant women was 2.2 times in high fluoride endemic areas.

Tea consumption was high in both districts, whereas tobacco/areca nut usage was high (55%) in the Nagaur district. It was observed that the consumption of black salt was around 38% in Nagaur and 28% in the Jodhpur district, and the consumption of churan/chat masala was 35.4% and 14.6% in Nagaur and Jodhpur districts, respectively. Table 4 shows drug consumption and selected dietary habits of pregnant women.

**Table 4.** Drug consumption and dietary habits of pregnant women.

Drug/Diet	Nagaur (%)	Jodhpur (%)
**Drugs**		
Anti-Depressant	3 (0.4)	28 (3.9)
Anti-infective	16 (2.2)	37 (5.1)
NSAID	23 (2.2)	25 (3.5)
Corticosteroids	1 (0.1)	0 (0)
Sedative	6 (0.8)	10 (1.4)
Ani-Emetic	159 (22.1)	54 (7.5)
Other like thyroid, DM	51 (7.1)	42 (5.8)
Iron tab	376 (52.2)	134 (18.6)
Calcium	376 (52.2)	131 (18.2)
Multivitamin	37 (5.1)	48 (6.7)
Homoeopathic	13 (1.8)	38 (5.3)
Ayurvedic	4 (0.6)	38 (5.3)
Albendazole	6 (0.8)	38 (5.3)
Vaccine T1 T2	376 (52.2)	40 (5.6)
Others Drugs	28 (3.9)	76 (10.6)
**Diet**		
Black salt	204 (28.3)	273 (37.9)
Churan/chat masala	105 (14.6)	255 (35.4)
Tea	495 (68.8)	628 (87.2)
Coca/coffee	39 (5.4)	40 (5.6)
Preserved fruits juice	48 (6.7)	48 (6.7)
Meat/canned fish	38 (5.3)	207 (28.7)
Dry fruits/walnut	61 (8.5)	41 (5.7)
Tobacco/Arecanut	43 (6)	396 (55.0)
Milk product	663 (92.1)	305 (42.4)
Eggs	46 (6.4)	90 (12.5)
Pickle	126 (17.5)	199 (27.6)
Fruits	622 (86.4)	427 (59.3)
Vegetable	699 (97.1)	676 (93.9)
Cereals	704 (97.8)	688 (95.6)
Pulses	688 (95.6)	677 (94.0)
Fat oils	692 (96.1)	667 (92.6)
Sugar/honey/Jaggery	664 (92.2)	77 (10.7)

## Discussion

Fluorosis is an endemic disease caused by excessive fluoride exposure from various sources. The element fluorine is a potent enzyme inhibitor, hormone disruptor, and neurotoxic, making it a double-edged sword that causes metabolic derangements ( Susheela *et al.*, 2018). Fluorosis manifests as a “linked disease” in many emerging Asian and African countries ( Susheela and Toteja, 2018). In many countries throughout the world, high fluoride level in groundwater has become one of the most serious health-related geo-environmental challenges ( Demelash *et al.*, 2019; Johnston and Strobel, 2020). Fluorosis has affected 21 Indian states. The Fluorosis Research and Rural Development Foundation, India survey mentioned that more than 66 million people are affected by fluorosis in India ( Susheela and Toteja, 2018).

The Bureau of Indian Standards (BIS) has specified fluoride levels in drinking water standards, with a maximum acceptable limit of 1.0 mg/L and an allowed limit of 1.5 mg/L in the absence of an alternative source ( Puri and Kumar, 2012). In some areas of Rajasthan, people drink water with fluoride levels as high as 24 mg/l ( Department of Environmental Engineering, Delhi Technological University, India *et al.*, 2014). In our study, mean fluoride values in water samples were 0.57 (range 0.0 to 2.7 PPM) in Jodhpur and 0.7 (range 0.0 to 3.4 PPM) in Nagaur. A similar study was carried out in the Punjab district of Faridkot, India. The mean value of water fluoride content was discovered to be 2.4 mg/L, which was higher than the allowable limits ( Goyal *et al.*, 2020). Hence, this region’s exposure to fluoride from drinking water is of considerable concern.

Even though low haemoglobin levels are frequently detected in clinical settings, they are rarely considered significant unless they are associated with the detection of a serious blood disease ( Andezhath, 2010). The greatest concern is that ingesting fluoride (F–) through water and food could result in serious health consequences. In our research, there was a significant association between fluoride levels in water and anaemia (p = 0.00001). The highly detrimental F– damages the lining of the intestine and hinders nutritional absorption when it enters the body through food, water, habit-forming chemicals, and dental products ( Susheela *et al.*, 2018). Hillman *et al.* discovered anaemia in cattle due to F– poisoning and fluorosis in the 1980s ( Hillman *et al.*, 1979). Excessive fluoride may affect brain development *in utero* and lead to neurological abnormalities, according to evidence from several human studies ( Grandjean, 2019; Guth *et al.*, 2020).

Urine has long been recognized as a significant diagnostic marker for fluoride intake since it is widely used, relatively easy to collect, and non-invasive ( Idowu *et al.*, 2019; Kumar *et al.*, 2017). In our study, pregnant women’s excretion of fluoride in urine and haemoglobin (Hb) had a statistically significant linear relationship. The direction of the relationship is negative, i.e., urine fluorosis and haemoglobin of pregnant women are negatively correlated. Excretion of mean fluoride value in the urine of pregnant females was found to be 1.4 (range from 0.0 to 12.7 mg/L) in Jodhpur district and 2.2 (range from 0.1 to 13.7 mg/L) in Nagaur district. Females with high urine fluoride levels have a higher risk of pregnancy difficulties such as anaemia and poor fetal outcomes, according to a study by Goyal *et al.* (2020).

According to AK Susheela *et al.*, drinking fluoride-free water and eating fluoride-free food reduced preterm births by four times and LBW babies by two times ( Susheela and Toteja, 2018).

In industrialized and developing countries, the burden of poor pregnancy outcomes (APOs), including preterm deliveries and LBWs, is significant ( Blencowe *et al.*, 2012; Lawn *et al.*, 2006; Lee *et al.*, 2013). Maternal anaemia was identified as a risk factor for low/insufficient birth weight in the current study, which was similar to findings from a prior study by Räisänen *et al.* (2014).

In India, anaemia affects 55.3 per cent of women aged 15 to 49 years old ( Kulasekaran, 2012). Anaemia in women during pregnancy has been linked to underweight children. Mother and newborn mortality rates in India are still high due to poor haemoglobin levels. Furthermore, anaemic mothers frequently give birth to low-weight newborns (as low as 1.0 to 1.2 kg), with a high risk of developmental problems or infant mortality later in life ( Andezhath, 2010).

A study finds that maternal haemoglobin (Hb > 110 g/l) protects against the risk of APOs in the study populations, which is consistent with prior findings ( Padhi *et al.*, 2015). With anaemia in the first or second trimester, a recent systematic review and meta-analysis found a significantly greater risk of LBW (OR: 1.29; 95 percent CI: 1.09–1.53) and preterm birth (OR: 1.21; 95 percent CI: 1.13–1.30) ( Haider *et al.*, 2013). Preterm delivery and LBW are important factors of child survival, impairments, stunting, and long-term negative implications for the beginning of non-communicable diseases later in life. They necessitate effective public health interventions.

The precise mechanism that links maternal anaemia to LBW remains unknown. Only a few prospective cohort studies have examined the relationships between maternal anaemia and LBW ( Bakacak *et al.*, 2015; Figueiredo *et al.*, 2019; Haider *et al.*, 2013; Rahman *et al.*, 2016). In Jodhpur, around 19 percent of newborns had a LBW, whereas, in Nagaur, nearly 22 percent had a LBW. According to another study, the relative risk of giving birth to LBW babies is 37% among the women with low haemoglobin levels ( Figueiredo *et al.*, 2019).

According to other studies, factors such as diet, excessive tea consumption, other associated nutritional deficiencies, and the use of fluoridated toothpaste have also been linked to the occurrence and severity of fluorosis ( Rao and Mahajan, 1990; Shruthi and Anil, 2018). The participants in our study mostly ate locally grown vegetarian cuisine daily and had a history of drinking tea and brushing their teeth with fluoridated toothpaste. In our study, there was a significant link between dental fluorosis and birth weight and anaemia (p > 0.005). Tea consumption was high in both districts, whereas tobacco/areca nut consumption was high (55 percent) in Nagaur. Black salt consumption was found to be roughly 38 percent in Nagaur and 28 percent in the Jodhpur district. In comparison, churan/chat masala consumption was found to be 35.4 percent and 14.6 percent in Nagaur and Jodhpur districts, respectively.

The prevalence of dental fluorosis was 13.17 percent in the study by Shruti *et al.*, with a high fluoride level ( Shruthi and Anil, 2018). Fluorosis is irreversible and only develops when the enamel is growing and exposed to fluoride. Fluoridated enamel is porous (objectionable secondary staining is common) and opaque rather than being a standard creamy-white translucent tint ( Mahantesha *et al.*, 2016). In our study, a significant association was also seen between fluoride level in the water and dental fluorosis (p = 0.00001) and baby birth weight (p = 0.000012) and LBW (p = 0.00001).

The presence of more than eight ppm of fluoride in drinking water over a long period might cause skeletal fluorosis ( Reddy, 2009). As a result of the persistent poisoning, bone density gradually increases, and joints stiffen and become painful.

Susheela *et al.*’s findings support the notion that iron and folic acid at current levels is beneficial in reducing the risk of LBW when combined with proper nutrition and reduced F– intake ( Susheela *et al.*, 2010). According to one study, efforts to raise Hb by iron and folic acid (IFA) supplementation through State and National Programs have yet to provide desired outcomes ( Beard, 2000; Vijayaraghavan *et al.*, 1990).

The use of validated instruments by previously trained researchers strengthens the internal validity of this study. A large sample size aids in extrapolating study findings to populations with similar geographies. In terms of the study’s weaknesses, the self-reported data may have resulted in calibration bias. Also, sample losses owing to missed follow-ups are a possibility.

With a coordinated effort to implement proper rules and procedures, the Millennium Development Goal of reducing early childhood mortality by two-thirds will be easier to attain ( “Global nutrition targets 2025,” n.d.). Here are a few solutions that have been presented for better implementation.

In every policy design, Behavior Change Communication (BCC) intervention must be an integral part that focuses on each stakeholder ( “tagged_ifas_participants_manual_for_healthcare_providers.pdf,” n.d.). Information, Education and Communication (IEC) initiatives must include educating pregnant women on the role of Hb and LBW children, as well as the do’s and don’ts of fluoride consumption in the diet, through media campaigns ( “Dietary guidelines for Indians.pdf,” n.d.; Goyal *et al.*, 2020). The role of dieticians and nutritionists must be escalated during ANC visits ( Kominiarek and Rajan, 2016). Ascorbic acid improves iron absorption (vitamin C). This should be stressed throughout the diet counselling session, which includes a list of vital fruits and vegetables that contain vitamin C that should be consumed ( Hallberg *et al.*, 1989). Iron absorption is inhibited by phytic acid/sodium phytate in brown bread and tannin in tea. The resulting vitamin B12 and folic acid deficiencies may cause anaemias. Vitamin B12 is not often investigated but is a key constituent for Hb biosynthesis ( Delimont *et al.*, 2017). Withdrawing fluoride from the diet may have repaired damage to the gastrointestinal mucosa/loss of microvilli, allowing nutrients to be absorbed, resulting in increased Hb and correction of anaemias ( Johnston and Strobel, 2020). This calls for assessing the effectiveness of de-fluoridation activities along with the most common food practices leading to high fluoride levels in pregnant women.

Fluoride assessment in body fluids is the primary criterion to know how much fluoride has entered the body. Regular monitoring of 24-hour urine samples and plasma samples may help researchers better understand the harmful effects of excessive fluoride exposure during pregnancy ( Goyal *et al.*, 2020). Thus, a monitoring and impact assessment must be part and parcel of future research related to fluoride and anaemic mothers.

In addressing the potential impact of various risk factors on anemia in the studied areas, we took measures to mitigate bias. Although the mean fluoride levels were not exceptionally high in both regions, we conducted a comprehensive assessment of potential contributors to anemia in pregnant women. This included the measurement of fluoride levels in both water and urine, acknowledging their role as risk factors.

Additionally, we investigated other factors such as dietary patterns, excessive consumption of tea and black salt, concomitant nutritional deficiencies, and the use of fluoridated toothpaste. We also considered the source of drinking water, aiming to account for any potential effects on hemoglobin levels in pregnant women. The study also delved into iron and folate supplementation for pregnant women, exploring the correlation with the risk of low birth weight in both districts. However, there was no significant difference in LBW babies in both districts (p=0.151).

It’s important to note that the study has its limitations, and while we addressed a few risk factors, there may be several area-specific that were not thoroughly explored, contributing to potential bias in the assessment of anemia in pregnant women.

## Limitations

The confounding factors included the exposure time to fluoride in drinking water, as well as the exposure to fluoride in age, obstetric history, supplements, diet, air, medication, water sources, cooking vessel etc. To minimize the confounding factors, we could have applied regression analysis to observe the impact of fluoride on maternal and child health. Further, recruitment of larger sample participants in a randomized manner could help in getting the impact of Fluoride on pregnant women.

However, we have collected data on variables like 24-hr diet recall to track nutrition aspects, past obstetric history, cooking vessels, medication, water sources and other co-morbidity like blood pressure, diabetes etc. But we could not perform analysis to see their impact on MCH in fluoride endemic areas.

## Conclusion

In fluoride endemic areas, other factors should be included besides iron and folic acid supplementation for improving anaemia in pregnant women. This calls for an assessment of the effectiveness of de-fluoridation activities along with the most common indigenous food practices in the area. In India, the role of fluoride as a risk factor in pregnancy has not been documented. In this context, the current study has added a significant body of knowledge that can be further scaled up.

## Data Availability

Zenodo: Jodhpur_Data File_anemia in Pregnancy_CSV.csv (Data of study participants from Jodhpur district on sociodemographic details, dietary habits and food consumption, addictions, drugs and medications consumed, drinking water sources, diseases, health parameters and anthropometric details) https://doi.org/10.5281/zenodo.7928263 ( Patil, 2023c). Zenodo: Nagaur_Data File_Anemia in Pregnancy_CSV.csv (Data of study participants from Nagaur district on sociodemographic details, dietary habits and food consumption, addictions, drugs and medications consumed, drinking water sources, diseases, health parameters and anthropometric details) https://doi.org/10.5281/zenodo.7928230 ( Patil, 2023d). Zenodo: Fluoride_studyQuestionnaire.pdf (Copy of blank Questionnaire used for data collection) https://doi.org/10.5281/zenodo.7962221 ( Patil, 2023b). Zenodo: STROBE checklist for ‘Study of anaemia in pregnancy and low birth weight in fluoride endemic area of Western Rajasthan: A cohort study’. https://doi.org/10.5281/zenodo.7780105 ( Patil, 2023a). Data are available under the terms of the Creative Commons Attribution 4.0 International license (CC-BY 4.0).

## Software availability

ArcGIS is a licensed online geographic information system software developed and maintained by Esri ( https://www.arcgis.com/index.html). The alternative software with the similar function is QGIS (QGIS is a free and open-source cross-platform desktop geographic information system application that supports viewing, editing, printing, and analysis of geospatial data. It can be downloaded through the link: https://qgis.org/).

## References

[ref1] Alemayehu GM Chernet AG Dumga KT : Determinants of Child Size at Birth and Associated Maternal Factor in Gurage Zone. 2020;21:8. PMC7253936 32500017

[ref2] Andezhath S : Anemia in pregnancy: An easily rectifiable problem. *Fluoride.* 2010;43:104–107.

[ref3] ArcGIS 10.3: The Next Generation of GIS Is Here. n.d.(accessed 5.25.23). ArcGIS Blog. Reference Source

[ref4] Arun AK Rustveld L Sunny A : Association between Water Fluoride Levels and Low Birth Weight: National Health and Nutrition Examination Survey (NHANES) 2013–2016. *IJERPH.* 2022;19:8956. 10.3390/ijerph19158956 35897326 PMC9332356

[ref5] Azami-Aghdash S Ghojazadeh M Azar FP : Fluoride Concentration of Drinking Waters and Prevalence of Fluorosis in Iran: A Systematic Review. 2013;7:7. 10.5681/joddd.2013.001 PMC3593199 23486733

[ref6] Bakacak M Avci F Ercan O : The effect of maternal hemoglobin concentration on fetal birth weight according to trimesters. *J. Matern. Fetal Neonatal Med.* 2015;28:2106–2110. 10.3109/14767058.2014.979149 25338012

[ref7] Batista Filho M Souza AI de Bresani CC : Anemia como problema de saúde pública: uma realidade atual. *Ciênc. Saúde Coletiva.* 2008;13:1917–1922. 10.1590/S1413-81232008000600027 18833369

[ref8] Beard JL : Effectiveness and strategies of iron supplementation during pregnancy. *Am. J. Clin. Nutr.* 2000;71:1288S–1294S. 10.1093/ajcn/71.5.1288s 10799404

[ref9] Beck S Wojdyla D Say L : The worldwide incidence of preterm birth: a systematic review of maternal mortality and morbidity. *Bull. World Health Org.* 2010;88:31–38. 10.2471/BLT.08.062554 20428351 PMC2802437

[ref69] Bello LD : Fluorosis: an ongoing challenge for India. Lancet Planet. Health. 2020;4:e94–e95. 10.1016/S2542-5196(20)30060-7 32220677 PMC7156153

[ref10] Blencowe H Cousens S Oestergaard MZ : National, regional, and worldwide estimates of preterm birth rates in the year 2010 with time trends since 1990 for selected countries: a systematic analysis and implications. *Lancet.* 2012;379:2162–2172. 10.1016/S0140-6736(12)60820-4 22682464

[ref11] Das TK Susheela AK Gupta IP : Toxic Effects of Chronic Fluoride Ingestion on the Upper Gastrointestinal Tract. *J. Clin. Gastroenterol.* 1994;18:194–199. 10.1097/00004836-199404000-00004 8034913

[ref12] Delimont NM Haub MD Lindshield BL : The Impact of Tannin Consumption on Iron Bioavailability and Status: A Narrative Review. *Curr. Dev. Nutr.* 2017;1:1–12. 10.3945/cdn.116.000042 29955693 PMC5998341

[ref13] Demelash H Beyene A Abebe Z : Fluoride concentration in ground water and prevalence of dental fluorosis in Ethiopian Rift Valley: systematic review and meta-analysis. *BMC Public Health.* 2019;19:1298. 10.1186/s12889-019-7646-8 31619212 PMC6794803

[ref14] Department of Environmental Engineering, Delhi Technological University, India: Jain A Singh S : Prevalence of Fluoride in Ground Water in Rajasthan State: Extent, Contamination Levels And Mitigation. *WPT.* 2014;2014:50–57. 10.15764/WPT.2014.02006

[ref15] Dhar V Bhatnagar M : Physiology and toxicity of fluoride. *Indian J. Dent. Res.* 2009;20:350. 10.4103/0970-9290.57379 19884722

[ref16] Dietary guidelines for Indians.pdf:n.d.

[ref17] Eshete A Alemu A Zerfu TA : Magnitude and Risk of Dying among Low Birth Weight Neonates in Rural Ethiopia: *A Community-Based Cross-Sectional Study.* *Int. J. Pediatr.* 2019;2019:1–8. 10.1155/2019/9034952 31223314 PMC6541952

[ref18] Fagin D : Second thoughts about fluoride. *Sci. Am.* 2008;298:74–81. 10.1038/scientificamerican0108-74 18225698

[ref20] Figueiredo ACMG Gomes-Filho IS Batista JET : Maternal anemia and birth weight: A prospective cohort study. *PLoS One.* 2019;14:e0212817. 10.1371/journal.pone.0212817 30884493 PMC6422668

[ref22] Gedalia I Brzezinski A Zukerman H : Placental Transfer of Fluoride in the Human Fetus at Low and High F-Intake. *J. Dent. Res.* 1964;43:669–671. 10.1177/00220345640430050801 14197051

[ref70] Goodman C Hall M Green R : Maternal fluoride exposure, fertility and birth outcomes: The MIREC cohort. Environ. Adv. 2022;7: 100135. 10.1016/j.envadv.2021.100135 PMC9837859 36644332

[ref23] Global nutrition targets 2025: low birth weight policy brief [WWW Document]:n.d.(accessed 11.23.22). Reference Source

[ref24] Goyal L Bakshi D Arora J : Assessment of fluoride levels during pregnancy and its association with early adverse pregnancy outcomes. *J. Family Med. Prim. Care.* 2020;9:2693–2698. 10.4103/jfmpc.jfmpc_213_20 32984109 PMC7491833

[ref27] Grandjean P : Developmental fluoride neurotoxicity: an updated review. *Environ. Health.* 2019;18:110. 10.1186/s12940-019-0551-x 31856837 PMC6923889

[ref28] Guth S Hüser S Roth A : Toxicity of fluoride: critical evaluation of evidence for human developmental neurotoxicity in epidemiological studies, animal experiments and in vitro analyses. *Arch. Toxicol.* 2020;94:1375–1415. 10.1007/s00204-020-02725-2 32382957 PMC7261729

[ref29] Haider BA Olofin I Wang M : Anaemia, prenatal iron use, and risk of adverse pregnancy outcomes: systematic review and meta-analysis. *BMJ.* 2013;346:f3443–f3443. 10.1136/bmj.f3443 23794316 PMC3689887

[ref31] Hallberg L Brune M Rossander L : The role of vitamin C in iron absorption. *Int. J. Vitam. Nutr. Res.* 1989;Suppl 30:103–108. 2507689

[ref32] Hillman D Bolenbaugh DL Convey EM : Hypothyroidism and Anemia Related to Fluoride in Dairy Cattle. *J. Dairy Sci.* 1979;62:416–423. 10.3168/jds.S0022-0302(79)83261-0 447892

[ref33] Idowu OS Azevedo LB Valentine RA : The use of urinary fluoride excretion to facilitate monitoring fluoride intake: A systematic scoping review. *PLoS One.* 2019;14:e0222260. 10.1371/journal.pone.0222260 31509581 PMC6738609

[ref34] Johnston NR Strobel SA : Principles of fluoride toxicity and the cellular response: a review. *Arch. Toxicol.* 2020;94:1051–1069. 10.1007/s00204-020-02687-5 32152649 PMC7230026

[ref36] Kapil U Kapil R Gupta A : National Iron Plus Initiative: Current status & future strategy. *Indian J. Med. Res.* 2019;150:239–247. 10.4103/ijmr.IJMR_1782_18 31719294 PMC6886130

[ref37] Kominiarek MA Rajan P : Nutrition Recommendations in Pregnancy and Lactation. *Med. Clin. North Am.* 2016;100:1199–1215. 10.1016/j.mcna.2016.06.004 27745590 PMC5104202

[ref38] Kulasekaran R : Influence of mothers’ chronic energy deficiency on the nutritional status of preschool children in Empowered Action Group states in India. *Int J Nutr Pharmacol Neurol Dis.* 2012;2:198. 10.4103/2231-0738.99471

[ref39] Kumar S Lata S Yadav J : Relationship between water, urine and serum fluoride and fluorosis in school children of Jhajjar District, Haryana, India. *Appl Water Sci.* 2017;7:3377–3384. 10.1007/s13201-016-0492-2

[ref40] Lawn JE Wilczynska-Ketende K Cousens SN : Estimating the causes of 4 million neonatal deaths in the year 2000. *Int. J. Epidemiol.* 2006;35:706–718. 10.1093/ije/dyl043 16556647

[ref41] Lee AC Katz J Blencowe H : National and regional estimates of term and preterm babies born small for gestational age in 138 low-income and middle-income countries in 2010. *Lancet Glob. Health.* 2013;1:e26–e36. 10.1016/S2214-109X(13)70006-8 25103583 PMC4221634

[ref42] Mahantesha T Dixit UB Nayakar RP : Prevalence of Dental Fluorosis and associated Risk Factors in Bagalkot District, Karnataka, India. *Int. J. Clin. Pediatr. Dent.* 2016;9:256–263. 10.5005/jp-journals-10005-1373 27843259 PMC5086015

[ref71] Malin AJ Busgang SA Garcia JC : Fluoride Exposure and Age of Menarche: Potential Differences Among Adolescent Girls and Women in the United States. Expo Health. 2022;14:733–742. 10.1007/s12403-021-00448-y

[ref43] McNeil AR Stanford PE : Reporting Thyroid Function Tests in Pregnancy 18. n.d. PMC4758281 26900190

[ref44] Padhi BK Baker KK Dutta A : Risk of Adverse Pregnancy Outcomes among Women Practicing Poor Sanitation in Rural India: A Population-Based Prospective Cohort Study. *PLoS Med.* 2015;12:e1001851. 10.1371/journal.pmed.1001851 26151447 PMC4511257

[ref45] Patil M : STROBE-cohort_checklist_Anemia in Pregnancy.docx. 2023a. 10.5281/zenodo.7780105

[ref46] Patil M : Fluoride_studyQuestionnaire.pdf. 2023b. 10.5281/zenodo.7962221

[ref47] Patil M : Jodhpur_Data File_anemia in Pregnancy_CSV.csv. 2023c. 10.5281/zenodo.7928263

[ref48] Patil M : Nagaur_Data File_Anemia in Pregnancy_CSV.csv. 2023d. 10.5281/zenodo.7928230

[ref49] Puri A Kumar M : A review of permissible limits of drinking water. *Indian J. Occup. Environ. Med.* 2012;16:40–44. 10.4103/0019-5278.99696 23112507 PMC3482709

[ref51] Rahman MM Abe SK Rahman MS : Maternal anemia and risk of adverse birth and health outcomes in low- and middle-income countries: systematic review and meta-analysis. *Am. J. Clin. Nutr.* 2016;103:495–504. 10.3945/ajcn.115.107896 26739036

[ref52] Räisänen S Kancherla V Gissler M : Adverse Perinatal Outcomes Associated with Moderate or Severe Maternal Anaemia Based on Parity in Finland during 2006-10: Anaemia and perinatal outcomes. *Paediatr. Perinat. Epidemiol.* 2014;28:372–380. 10.1111/ppe.12134 24938307

[ref53] Rao KV Mahajan CL : Fluoride content of some common South Indian foods and their contribution to fluorosis. *J. Sci. Food Agric.* 1990;51:275–279. 10.1002/jsfa.2740510215

[ref54] Reddy D : Neurology of endemic skeletal fluorosis. *Neurol. India.* 2009;57:7–12. 10.4103/0028-3886.48793 19305069

[ref72] Ren C Zhang P Yao X-Y : The cognitive impairment and risk factors of the older people living in high fluorosis areas: DKK1 need attention. BMC Public Health. 2021;21:2237. 10.1186/s12889-021-12310-6 34886821 PMC8656079

[ref55] Sachdev HPS : LOW BIRTH WEIGHT IN SOUTH ASIA. 2001;21:19.

[ref56] Shruthi M Anil N : A comparative study of dental fluorosis and non-skeletal manifestations of fluorosis in areas with different water fluoride concentrations in rural Kolar. *J Family Med Prim Care.* 2018;7:1222–1228. 10.4103/jfmpc.jfmpc_72_18 30613501 PMC6293885

[ref59] Susheela AK : Dental Fluorosis and its Extended Effects. *Indian J. Pediatr.* 2013;80:715–717. 10.1007/s12098-013-1100-1 23801348

[ref60] Susheela AK Mondal NK Gupta R : Effective interventional approach to control anaemia in pregnant women. *Curr. Sci.* 2010;98:11.

[ref62] Susheela AK Mondal NK Gupta R : Fluorosis is Linked to Anaemia. *Curr. Sci.* 2018;115:692. 10.18520/cs/v115/i4/692-700

[ref63] Susheela AK Toteja GS : Prevention & control of fluorosis & linked disorders: Developments in the 21st Century - Reaching out to patients in the community & hospital settings for recovery. *Indian J. Med. Res.* 2018;148:539–547. 10.4103/ijmr.IJMR_1775_18 30666981 PMC6366265

[ref64] tagged_ifas_participants_manual_for_healthcare_providers.pdf:n.d.

[ref65] Vijayaraghavan K Brahmam GNV Nair KM : Evaluation of national nutritional anemia prophylaxis programme. *Indian J. Pediatr.* 1990;57:183–190. 10.1007/BF02722084 2246014

[ref66] Watkins WJ Kotecha SJ Kotecha S : All-Cause Mortality of Low Birthweight Infants in Infancy, Childhood, and Adolescence: Population Study of England and Wales. *PLoS Med.* 2016;13:e1002018. 10.1371/journal.pmed.1002018 27163787 PMC4862683

[ref67] WHA Global Nutrition Targets 2025: Low Birth Weight Policy Brief:n.d.

[ref68] World Health Organization: *The global prevalence of anaemia in 2011.* Geneva: World Health Organization;2015.

