# Supplementary material for: Study of anaemia in pregnancy and low birth weight in fluoride endemic area of Western Rajasthan: a cohort study
Source: F1000Res. 2024 Mar 19;12:602. Originally published 2023 Jun 5. [Version 2] doi: 10.12688/f1000research.134720.2 (PMC10822042; doi:10.12688/f1000research.134720.2)
Supplement: Peer review history

# Reviewer report: Reviewer response for version 2

**DOI:** 10.5256/f1000research.163694.r257331

**Published:** 2024-04-04

**Authors:** Rungrote Natesirinilkul

**Affiliations:** 1 Chiang Mai University and Maharaj Nakorn Chiang Mai Hospital, Chiang Mai, Thailand

I am pleased with the responses of the authors. I have no more comment to add.

Is the work clearly and accurately presented and does it cite the current literature?

Yes

If applicable, is the statistical analysis and its interpretation appropriate?

Partly

Are all the source data underlying the results available to ensure full reproducibility?

Partly

Is the study design appropriate and is the work technically sound?

Yes

Are the conclusions drawn adequately supported by the results?

Partly

Are sufficient details of methods and analysis provided to allow replication by others?

Yes

Reviewer Expertise:

anemia, bleeding disorders, thrombosis, blood transfusion

I confirm that I have read this submission and believe that I have an appropriate level of expertise to confirm that it is of an acceptable scientific standard.

# Reviewer report: Reviewer response for version 1

**DOI:** 10.5256/f1000research.147800.r179723

**Published:** 2024-01-27

**Authors:** Apurvakumar Pandya

**Affiliations:** 1 Parul Institute of Public Health, Parul University, Vadodara, Gujarat, India

The study is well carried out and nicely written.

 This prospective cohort study attempted to identify linkages between fluorosis and the low-birth weight of newborn babies with anaemic mothers.  In this study, Antenatal mothers until the 20th week of gestation were followed up till delivery in the Antenatal Clinic of a  fluoride-endemic district  and not-so-endemic district of Western Rajasthan.

 Considering current scenario, this study provides important insights. As this manuscript is well written, I have no comments for improvement.

Is the work clearly and accurately presented and does it cite the current literature?

Yes

If applicable, is the statistical analysis and its interpretation appropriate?

Yes

Are all the source data underlying the results available to ensure full reproducibility?

Yes

Is the study design appropriate and is the work technically sound?

Yes

Are the conclusions drawn adequately supported by the results?

Yes

Are sufficient details of methods and analysis provided to allow replication by others?

Yes

Reviewer Expertise:

public health, anemia control, health behaviour change, public mental health, health technology assessment

I confirm that I have read this submission and believe that I have an appropriate level of expertise to confirm that it is of an acceptable scientific standard.

# Response

**Published:** 2024-01-27

**Authors:** MANOJ PATIL

**Affiliations:** Datta Meghe Institute of Higher Education and Research, Wardha, India

Thank you very much for your appreciation. The authors are grateful for your timely response on this article and your valuable comments.

# Reviewer report: Reviewer response for version 1

**DOI:** 10.5256/f1000research.147800.r232742

**Published:** 2024-01-22

**Authors:** Rungrote Natesirinilkul

**Affiliations:** 1 Chiang Mai University and Maharaj Nakorn Chiang Mai Hospital, Chiang Mai, Thailand

This paper is interesting. However, a few issues are needed to be clarified more. 

 1.Please provide the exact mean number of hemoglobin level in each group apart from percentage of pregnant women with anemia. 

 2.Please provide the explanation why LBW neonates were more commonly found in Jodhpur district. 

 3.Several risk factors were different between both areas where this study was performed. How do we decrease the bias of the other risk factors causing anemia in this study as the mean levels of fluoride were not extremly high in both areas.

Is the work clearly and accurately presented and does it cite the current literature?

Yes

If applicable, is the statistical analysis and its interpretation appropriate?

Partly

Are all the source data underlying the results available to ensure full reproducibility?

Partly

Is the study design appropriate and is the work technically sound?

Yes

Are the conclusions drawn adequately supported by the results?

Partly

Are sufficient details of methods and analysis provided to allow replication by others?

Yes

Reviewer Expertise:

anemia, bleeding disorders, thrombosis, blood transfusion

I confirm that I have read this submission and believe that I have an appropriate level of expertise to confirm that it is of an acceptable scientific standard, however I have significant reservations, as outlined above.

# Response

**Published:** 2024-03-12

**Authors:** MANOJ PATIL

**Affiliations:** Datta Meghe Institute of Higher Education and Research, Wardha, India

We are extremely thankful to the Reviewer for bringing new insights for improving the

 manuscript.

**Reviewer Comments**

**Response**

 1. Please provide the exact mean number of hemoglobin levels in each group apart from the percentage of pregnant women with anemia. 

**The mean Hb (gm%) level in pregnant women in the Jodhpur district was 10.4gm% as compared to the Nagaur district (9.1gm%).**

 2. Please provide an explanation why LBW neonates were more commonly found in the Jodhpur district

**In our study, the mean weight of newborns from the Jodhpur district was 2.8 ± 0.56 kg, and from Nagaur was 2.8 ± 0.44 kg.**

**Around 19% of newborns in Jodhpur and around 22% in Nagaur had LBW. However, there was no significant difference in LBW babies in both districts (p=0.151)**

 3. Several risk factors were different between both areas where this study was performed. How do we decrease the bias of the other risk factors causing anemia in this study as the mean levels of fluoride were not extremely high in both areas?

**In addressing the potential impact of various risk factors on anemia in the studied areas, we took measures to mitigate bias. Although the mean fluoride levels were not exceptionally high in both regions, we conducted a comprehensive assessment of potential contributors to anemia in pregnant women. This included the measurement of fluoride levels in both water and urine, acknowledging their role as risk factors.**

**Additionally, we investigated other factors such as dietary patterns, excessive consumption of tea and black salt, concomitant nutritional deficiencies, and the use of fluoridated toothpaste. We also considered the source of drinking water, aiming to account for any potential effects on hemoglobin levels in pregnant women. The study also delved into iron and folate supplementation for pregnant women, exploring the correlation with the risk of low birth weight in both districts. However, there was no significant difference in LBW babies in both districts (p=0.151)**

**It's important to note that the study has its limitations, and while we addressed a few risk factors, there may be several area-specific that were not thoroughly explored, contributing to potential bias in the assessment of anemia in pregnant women.**

# Reviewer report: Reviewer response for version 1

**DOI:** 10.5256/f1000research.147800.r179725

**Published:** 2023-07-05

**Authors:** Umesh Charantimath

**Affiliations:** 1 Department of Community Medicine, KLE Academy of Higher Education and Research J N Medical College, Belagavi, Karnataka, India

It is one of the nicely written manuscript. It would be very nice if you add recent articles in the Introduction part rather than quoting a three decade old article. Low birth weight has multiple causes and in this article you have tried to enlist them, but how to minimize the confounding factors and prioritize fluoride still needs to be explained in detail. 

 Fluorosis is a very important public health problem in the LMICs. In this article the authors have clearly mentioned the importance of fluorosis in relation to anemia in pregnancy and LBW. There are many other reasons for LBW and anemia in pregnant women. How the fluoride content in the drinking water will have an adverse impact on maternal and child health has tried to be explained by the authors. What the impact fluorosis has on other age groups has to be addressed.

 I feel larger sample size should be looked into and along with that the multiple variables also need to be collected such as nutrition, obstetric history, other comorbidities etc. to come to a final consensus about at what level the fluorosis will have an adverse impact on maternal and child health.

Is the work clearly and accurately presented and does it cite the current literature?

Yes

If applicable, is the statistical analysis and its interpretation appropriate?

Yes

Are all the source data underlying the results available to ensure full reproducibility?

Yes

Is the study design appropriate and is the work technically sound?

Yes

Are the conclusions drawn adequately supported by the results?

Yes

Are sufficient details of methods and analysis provided to allow replication by others?

Yes

Reviewer Expertise:

Maternal child health

I confirm that I have read this submission and believe that I have an appropriate level of expertise to confirm that it is of an acceptable scientific standard.

# Response

**Published:** 2023-07-06

**Authors:** MANOJ PATIL

**Affiliations:** Datta Meghe Institute of Higher Education and Research, Wardha, India

Respected Sir,

 Thank you very much for taking time out of your busy schedule for this review. Also we are obliged for your valuable comments and suggestions on this manuscript and for approving the article. We are extremely thankful for bringing new insights for improving the manuscript.

 Please see below the reply to your comments for further perusal-

**Comment -** **Recent articles in the Introduction**

**Response: Following are the recent references-**

 1. Fluorosis, an underestimated and sometimes debilitating condition, is being caused by high levels of fluoride in food and contaminated groundwater in India.

**Reference:**

 Del Bello, Lou. Fluorosis: an ongoing challenge for India

 The Lancet Planetary Health,2020. Volume 4, Issue 3, e94 - e95

 2. Interestingly, fluoride exposure during pregnancy was not significantly associated with fertility or birth outcomes, especially among women living in areas with levels of fluoride consistent with water fluoridation.

**Reference:**

 Goodman C, Hall M, Green R, Hornung R, Martinez-Mier EA, Lanphear B, Till C. Maternal fluoride exposure, fertility and birth outcomes: The MIREC cohort. Environ Adv. 2022 Apr;7:100135. doi: 10.1016/j.envadv.2021.100135. Epub 2021 Nov 2. PMID: 36644332; PMCID: PMC9837859.

 3. Contrastingly, other literatures have found increased risk of adverse birth outcomes in areas where fluoride levels in drinking water are high (> 1.5 mg/L)

**Reference:**

 Goyal LD, Bakshi DK, Arora JK, Manchanda A, Singh P. Assessment of fluoride levels during pregnancy and its association with early adverse pregnancy outcomes. J Family Med Prim Care. 2020 Jun 30;9(6):2693-2698. doi: 10.4103/jfmpc.jfmpc_213_20. PMID: 32984109; PMCID: PMC7491833.

**Comment:** **How to minimize the confounding factors and prioritize fluoride still need to be explored.**

**Response:**

 The confounding factors included the exposure time to fluoride in drinking water, as well as the exposure to fluoride in age, obstetric history, supplements, diet, air, medication, water sources, cooking vessel etc.

 To minimize the confounding factors, we could have applied regression analysis to observe the impact of fluoride on maternal and child health. Further, recruitment of larger sample participants in a randomized manner could help in getting the impact of Fluoride on pregnant women (Limitation of Study).

**Comment:** **Impact of Fluorosis on other age groups**

 It would be interesting to explore the impact of Fluoride on different age groups.

**Responses:**

**Adolescents-**

 A study conducted in the United States discovered that greater water fluoride concentrations were connected with a 3.3-month earlier age of menarche.

 Malin, A.J., Busgang, S.A., Garcia, J.C. et al. Fluoride Exposure and Age of Menarche: Potential Differences Among Adolescent Girls and Women in the United States. Expo Health 14, 733–742 (2022). https://doi.org/10.1007/s12403-021-00448-y

**Elderly-**

 Further, cognitive skills in older adults living in high fluoride drinking water locations may be compromised.

 Ren C, Zhang P, Yao XY, Li HH, Chen R, Zhang CY, Geng DQ. The cognitive impairment and risk factors of the older people living in high fluorosis areas: DKK1 need attention. BMC Public Health. 2021 Dec 9;21(1):2237. doi: 10.1186/s12889-021-12310-6. PMID: 34886821; PMCID: PMC8656079.

**Children-**

 A south Indian researcher discovered that nutritional status has an effect on the severity of fluorosis, with impoverished children being more affected by severe fluorosis.

 Mahantesha T, Dixit UB, Nayakar RP, Ashwin D, Ramagoni NK, Kamavaram Ellore VP. Prevalence of dental fluorosis and associated risk factors in Bagalkot District, Karnataka, India. Int J Clin Pediatr Dent. (2016) 9:256–63. doi: 10.5005/jp-journals-10005-1373

**Comment: A larger sample size should be looked in and along with multiple variables such as nutrition, obstetric history, other co-morbidities to come to a final consensus to see the impact of fluoride on MCH.**

**Response:**

 However, we have collected data on variables like 24-hr diet recall to track nutrition aspects, past obstetric history, cooking vessels, medication, water sources and other co-morbidity like Blood pressure, diabetes etc. but we could not perform analysis to see their impact on MCH in fluoride endemic areas (Limitation of the study).
